# Impact of community-based precision functional training on older adults’ walking and cognitive abilities in rural Taiwan: a pre–posttest study

**DOI:** 10.1186/s12877-024-05422-2

**Published:** 2024-10-11

**Authors:** Fang-Lin Kuo, Zih-Yong Liao, Pei-Chun Liao, Hsiu-Hua Wang

**Affiliations:** 1 National Center for Geriatrics and Welfare Research, National Health Research Institutes, 8, Xuefu W. Rd., Huwei Township, Yunlin County, 63247 Taiwan; 2 Department of Athletic Sports, National Chung Cheng University, Chiayi County, Taiwan

**Keywords:** Community, Older adults, Precision functional training, Mobility, Rural area

## Abstract

**Background:**

Functional training is essential for maintaining the independence of older adults, especially in rural areas with limited resources. In this study, we assess the short-term and long-term impacts of the Precision Functional Training (PFT) program on mobility outcomes, specifically walking performance, and cognitive ability in community-dwelling older adults. The unique feature of this training program was its countywide, community-based, and tailored approach, designed to strengthen the functional abilities of older adults living in rural areas.

**Methods:**

158 older adults aged 65 years and above were assessed in this one-group pre–posttest study conducted in 11 community care stations in Chiayi County, Taiwan. Participants had two follow-ups, with data collection on mobility and cognition. The 12-week PFT program, led by certified trainers, integrated aerobic, strength, and cognitive elements. Primary outcomes, including changes in gait, falls, and cognition, were analyzed using linear mixed effects and logit models.

**Results:**

Strengthening mobility is critical to slowing functional decline in older adults. The PFT program led to significant improvements in cognitive function and several gait parameters compared with the baseline. Participants with limited mobility showed enhanced activities of daily living 1-month post-training, but these gains did not persist at the 1-year mark. No significant differences were observed in fall occurrence and knee extension strength.

**Conclusions:**

The training did not have a long-term effect; thus, more frequent practice may be necessary. Risk assessment and community-based interventions, particularly for older adults with a higher risk of falls, are recommended. Future prospective randomized controlled trials are needed to evaluate the PFT program’s effectiveness in preserving mobility.

## Background

As Taiwan approaches becoming a super-aged society, preventing age-related physical decline and disabilities has become a key public health priority [1]. Aging often results in decreased strength, balance, flexibility, and endurance, essential for mobility and quality of life [2–4]. This decline in physical capabilities significantly impacts older adults, resulting in increased care costs, reduced independence, and higher mortality rates; these problems are particularly prevalent in Taiwan [5, 6]. Mobility, particularly the ability to walk, is essential for maintaining older adults’ independence. In Taiwan, a decline in walking ability begins to affect the population starting at around 60 years old [7]. Limited walking ability affects not only health and mortality but also contributes to social isolation, especially among older adults who live alone [8, 9]. Moreover, it has been a predictor of negative outcomes for older adults and poses a higher risk of falls, which are a significant concern [10]. Up to 34% of community-dwelling older adults experience falls annually, leading to injuries and fatalities [7, 11–13]. Falls further limit mobility and create a cycle of diminished physical activity [14]. Risk factors for falls include cognitive decline, living alone, use of walking aids, and limited engagement in physical activities [7, 15]. Sedentary behavior exacerbates these issues, being linked to negative gait outcomes, further compounding the problem [9, 16]. These factors reveal the need for targeted interventions, including physical activity programs and fall risk assessment.

Gait parameters such as gait speed or balance are clinically meaningful indicators of walking ability as they not only reflect the efficiency of movement but also correlate strongly with several health outcomes [6, 17]. Slower gait speeds or poor balance are associated with an increased risk of falls and cognitive decline, as well as higher mortality rates [6, 18]. It is easy for healthcare professionals to use measures to gain a comprehensive understanding of older adults’ functional capacity, mobility status, and potential need for interventions to improve or maintain their walking ability. In addition to gait analysis, muscle strength is commonly used to quantify weakness and decline in functional performance, such as walking, in older adults [19]. Knee extension strength, particularly, is considered sensitive in identifying changes in walking ability and serves as a reliable measure in assessing functional capacity [19–21]. Walking difficulty in older adults can be influenced by factors such as age, obesity, amount of physical activity, and living alone [7, 22]. Environmental factors such as multi-story houses without elevators in rural Taiwan may further limit mobility for older adults with walking difficulties [22].

Walking is a complex and highly skilled form of gait. Gait disorders are more prevalent in individuals with cognitive impairment [23]. Thus, there is a need to consider cognitive function when assessing walking ability, as cognitive impairment is strongly tied to gait abnormalities [23, 24]. Functional training that enhances both walking and cognitive function could improve overall abilities, strengthen lower extremities, enhance balance, and aid in preventing falls among older adults [25]. Dual-task exercises of varying intensities and that activate interconnected neural pathways have been shown to benefit both cognitive function and mobility [26, 27]. Therefore, functional training programs should incorporate cognitive exercises and address lower-extremity strength [25], making them more inclusive and adaptable to individuals with moderate mobility limitations.

Promoting physical activity is crucial for reducing walking decline and fall risks among community-dwelling older adults [28]. However, many functional training programs often lack consistency and evidence-based guidance, especially for community coordinators working with older adults. Current research on community group programs often focuses on younger individuals, favoring intense exercise and often excluding those using assistive walking devices, which limits practical applicability [29, 30]. Preventing the rapid onset of disability could reduce the future reliance on long-term care and emergency health services [31]. Community-based programs are proposed to prevent falls, particularly in those at higher risk owing to factors such as being female, living alone, and experiencing certain health conditions (e.g., urinary incontinence, perceived unhealthiness, and pain) [15]. While these programs can improve wellbeing and walking ability, disparities between rural and urban areas—driven by differences in service availability and accessibility—remain a challenge [32]. In Taiwan, community care stations are critical in supporting programs that foster interaction, reduce isolation [33, 34], and address the higher prevalence of cognitive decline [35, 36] in rural areas.

The community-based Precision Functional Training (PFT) program has been developed by an interdisciplinary team consisting of sports professionals, physical therapists, and educational researchers. This program provides a systematic approach for assessing, planning, and implementing group-tailored physical activity strategies for community-dwelling older adults. This approach enhances uniformity and flexibility in addressing older adults’ activities and reduces complications related to immobility. By integrating physical and cognitive training, the PFT program aims to improve coordination and functional capacity. Each session includes warm-up exercises, upper- and lower-extremity workouts, transitions between sitting and standing positions, and cognitive tasks. The program is designed to be adjustable and inclusive, accommodating participants with varying physical capacities, including those with walking difficulties or using assistive devices, thereby eliminating barriers and promoting inclusivity.

We evaluated the PFT program initiated by the Social Affairs Bureau of Chiayi County, an area with the highest proportion (21.4%) of older adults in Taiwan by 2022 [37]. Given the evident need for community care programs and health-promoting resources, we aimed to: (1) assess the impact of the 12-week PFT program, which integrated aerobic, strength, and cognitive elements, on functional outcomes related to cognition and walking in older adults and (2) analyze changes in gait, falls, and knee extension strength as primary outcomes, along with their related factors. This study provides insights into the development of targeted training programs to prevent the onset or progression of walking ability decline through assessment.

## Methods

### Participants and settings

The PFT program was initiated between April and May 2022 at 11 local community care stations. Recruitment was based on defined inclusion and exclusion criteria to ensure adequate cognitive and mobility capacity to understand and participate in the study. The inclusion criteria were (1) adults aged 65 years or older and (2) ability to walk independently, with or without the use of assistive devices. The exclusion criteria were (1) possible moderate-to-severe cognitive impairment and (2) individuals under guardianship, as they could not make independent decisions.

To ensure appropriate recruitment, communication with community care station managers (acting as gatekeepers) began 2 months before contacting potential participants. The managers initially screened individuals based on their cognitive and walking capacity based on their daily interaction, ensuring that only those competent to participate in the study were considered. Subsequently, the research team introduced the study to the selected participants individually. During this introduction, participants were screened further for cognitive capacity through a “teach-back” method, where they were asked two key questions: “What will we do during the study?” and “What are your rights to drop out, and how should you respond if you no longer want to continue?” This approach ensured that participants fully understood the study and their rights before enrolling.

Older adults who demonstrated adequate understanding and agreed to participate were then enrolled in the study. All 158 participants voluntarily provided informed consent after having adequate time to ask questions and consider their decision. Following consent, managers scheduled pretest appointments for the participants. These appointments included assessments such as walking and knee extension strength tests, along with on-site questionnaire interviews.

### PFT

The PFT program, designed by the Department of Athletic Sports of National Chung Cheng University, consisted of 12 sessions, each lasting 120 min, over 12 weeks. The program incorporated bodyweight exercises and supplemental aids targeting essential life functions, including pushing, pulling, lifting, jumping, throwing, and squatting. Led by certified trainers, the sessions adhered to the Frequency, Intensity, Time, and Type principles, gradually increasing exercise intensity and complexity over time. The Rated Perceived Exertion scale [38] was used to gauge participants’ perceived exercise load, with warm-up activities ranging from 3 to 5 points, cardiorespiratory endurance from 4 to 7 points, and muscle strength training from 5 to 7 points.

Cultural relevance was integrated into the program by using local languages (Taiwanese or Hakka) to create rhymes and incorporating older adults’ memories or songs into choreographed dances that simulated functional movements, such as planting rice seedlings. Interactive activities, such as ball tossing, were included to enhance engagement. The implementation followed the PRICE scheme: Progressive exercises tailored to individual needs, Relevance through integrating music and language, Interaction to foster engagement, Challenge by promoting self-efficacy and motivation, and Evaluation using health indicators to refine the program.

The program focused on improving participants’ functional capabilities and mobility and reducing fall risk by integrating physical, cognitive, and coordination elements. A key feature was dual-task training, where cognitive exercises (COGNICISE) were combined with physical activities, challenging participants to engage their minds and bodies simultaneously. Exercises included tasks such as counting backward and identifying odd or even numbers while moving, aimed at improving cognitive function and decision making in real-life scenarios. The course design was flexible, allowing trainers to adjust exercises while maintaining core principles, ensuring inclusivity for participants with varying cognitive or physical abilities.

**Fig. 1:**
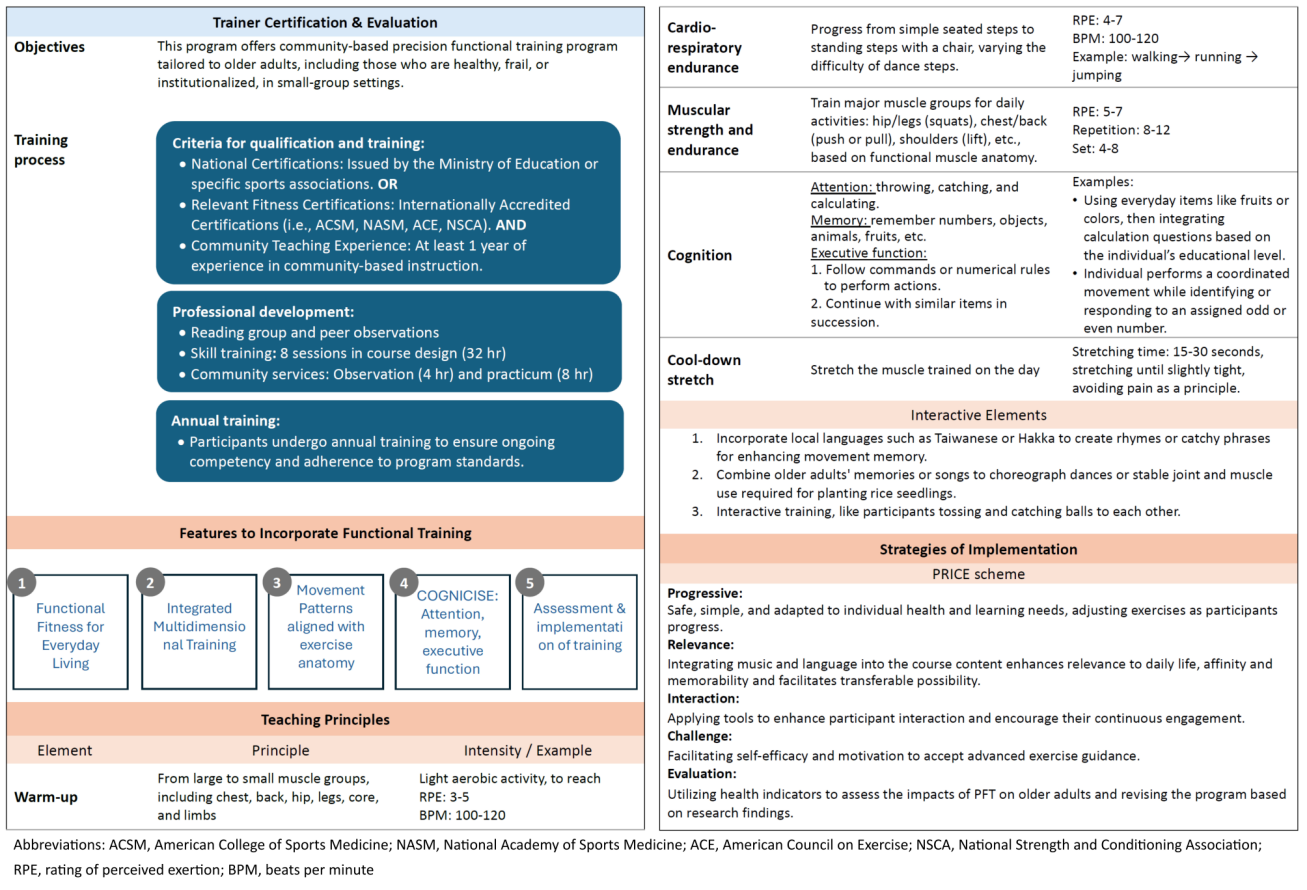
Framework of trainer qualifications and the Precision Functional Training program. *Abbreviations* ACSM, American College of Sports Medicine; NASM, National Academy of Sports Medicine; ACE, American Council on Exercise; NSCA, National Strength and Conditioning Association; RPE, rating of perceived exertion; BPM, beats per minute

Sessions concluded with a mildly responsive exercise and a cool-down segment to ensure a gradual return to a resting state. Throughout the program, trainers continuously assessed the group’s physical capabilities and adjusted the exercises as needed. For participants with walking difficulties or who used mobility devices, alternative instructions and modified exercises were provided, ensuring inclusivity and reducing barriers to participation. The integration of cognitive and physical exercises was not only designed to improve immediate functional capabilities but also to be straightforward and easy for participants to practice at home. This approach empowered participants to maintain and enhance their skills outside of the structured sessions, contributing to long-term benefits (Fig. 1).

### Trainer certification and evaluation

Professional trainers are trained by the Department of Athletic Sports at National Chung Cheng University. To qualify as a professional trainer, individuals must hold either a national certification issued by the Ministry of Education or specific sports associations in Taiwan, or relevant fitness certifications from internationally accredited organizations such as American College of Sports Medicine, National Academy of Sports Medicine, American Council on Exercise, or National Strength and Conditioning Association. In addition, trainers are required to have at least one year of experience in community-based instruction. This combination of certified expertise and practical experience ensures that trainers have a strong foundation in athletic or fitness education.

The training sessions integrate functional anatomy, communication and interaction, teaching methods and strategies, practicums, study groups, and evaluations. The study groups are particularly focused on reinforcing professional theories, ensuring a deep understanding of the material. Following the training, trainers undergo a rigorous evaluation process designed to assess their knowledge and its practical application. The evaluations include simulations of specific groups and scenarios, with a particular focus on practical skills for older adults. Trainers who successfully pass the examinations earn certificates and are placed in community care stations. Supported by the local government, the licensure rate for 2022 was 50%.

### Measures

Assessing mobility in older adults involves an array of walking-related indicators, including gait parameters, the timed up and go (TUG) test, knee extension strength, and fall occurrences. This multi-faceted approach provides a comprehensive perspective on participants’ mobility and associated risks.

In this study, participants completed a 6-meter walk equipped with a pager-sized accelerometer (Physilog; Gait Up, Lausanne, Switzerland). Physilog allows for the measurement of gait in various walking environments and has been effectively used to assess gait parameters in community-dwelling older adults, demonstrating its applicability for this population [39, 40]. Physilog exhibits good to excellent reliability (coefficients = 0.75–0.95) across various walking tasks and environments in middle-aged and older adults [41, 42]. Gait speed, a key predictor of health outcomes for walking limitations, falls, and cognitive decline, was measured [43]. A gait speed below 1.0 m/s typically indicates mobility limitations [43]. Maintaining a faster cadence and longer strides tend to result in a slower decline in walking ability [44].

The TUG test assessed mobility by measuring the time taken to transition from sitting to a standing position, walk 3 m, turn around, return to a chair, and sit down [45]. This test is recommended for identifying older adults with balance problems, fall risk, and frailty [46, 47]. A TUG score exceeding 13.5 s suggests a higher fall risk for older adults [46].

Leg strength, an indicator of lower-limb strength and essential for maintaining muscle mass and walking performance, was assessed through knee extension strength using a handheld dynamometer (Hoggan Scientific, microFET2, USA) [19, 48, 49]. The microFET2 has demonstrated good reliability and validity in measuring lower-limb muscle strength in adults (intraclass correlation coefficients ≥ 0.70) [50]. Studies have reported a wide range of knee extension strength values in community-dwelling older adults, with strength ranging from 0.35 to 0.45 kg/kg [19] and 21.3 to 29.77 kg-force [21]. In adults, the average strength ranges from 17.53 to 20.37 kg-force [50]. In this study, knee extension strength was measured thrice for the lower limb, with the highest score being selected for analysis.

Falls pose a significant concern for older adults, leading to severe injuries, loss of independence, and increased mortality risk. Self-reported fall histories were collected in this study, with multiple falls in the past year considered a higher risk for future falls and compromised mobility [51].

The Barthel index for activities of daily living (ADL) [52] and the Lawton instrumental activities of daily living (IADL) scale [53, 54] were assessed through self-reported data from participants, supplemented by consultations with personnel from the care station. ADL consist of 10 basic activities: feeding, grooming, bathing, dressing, bowel and bladder care, toilet use, ambulation, transfers, and stair climbing. The total score ranges from 0 to 100, with higher scores indicating greater levels of functional capacity [52]. IADL cover tasks such as using the telephone, shopping, food preparation, housekeeping, laundry, transportation, medication management, and handling finances, with a total score of 8; each activity contributes 1 point [53, 54].

Cognitive status was measured using the Mini-Cog tool [55], which integrates recall and clock drawing exercises. Scores on this tool range from 0 to 5, with scores of 0–2 indicating a high likelihood of cognitive impairment [55].

To understand the lifestyles of older adults, particularly their health-promoting behaviors throughout the week, the Short-Form Chinese Health-Promoting Lifestyle Profile [56] was used. This instrument comprises six lifestyle domains: self-accomplishment, responsibility for health, exercise, nutrition, social relationships, and stress management, encompassing 24 items [56]. The total score ranges from 24 to 96, with higher scores indicating more health-promoting behaviors. The tool has demonstrated reliability, with coefficients ranging from 0.63 to 0.79 in the adult population [56].

### Procedure for pretest and posttest

The PFT assessment followed a pretest–posttest framework, conducted in two stages: pre-program (baseline), and 1-month and 1-year post-program. This approach aimed to track changes in primary outcomes (gait parameters, fall incidents, and knee extension strength) attributable to the intervention.

The participants’ mobility status was assessed at baseline. They self-reported any falls, while isometric dynamometry measured knee strength. The posttest, occurring 1 month and 1 year after the 12-week program, repeated identical assessments to evaluate program benefits. Using identical protocols, the same trained personnel measured gait parameters, documented falls, and evaluated knee extension strength. A comparison of these two assessments revealed the program’s impact on mobility and cognitive performance, potentially enhancing gait, reducing falls, and boosting knee strength in older adults.

### Data analysis methods

To assess the program’s impact, we utilized linear mixed-effects and logit models to process repeated measures. Linear mixed-effects models were used to evaluate changes in continuous outcomes, such as gait parameters and knee extension strength. These models incorporated both fixed and random effects, accounting for the variations among participants. The incorporation of random effects enabled us to model the correlations among repeated measures, yielding more reliable outcomes. Logit models were used to analyze the incidence of falls as a categorical outcome. Analyses were performed using SAS 9.4 (SAS Institute, Cary, NC, USA).

## Results

### Participant characteristics

**Fig. 2:**
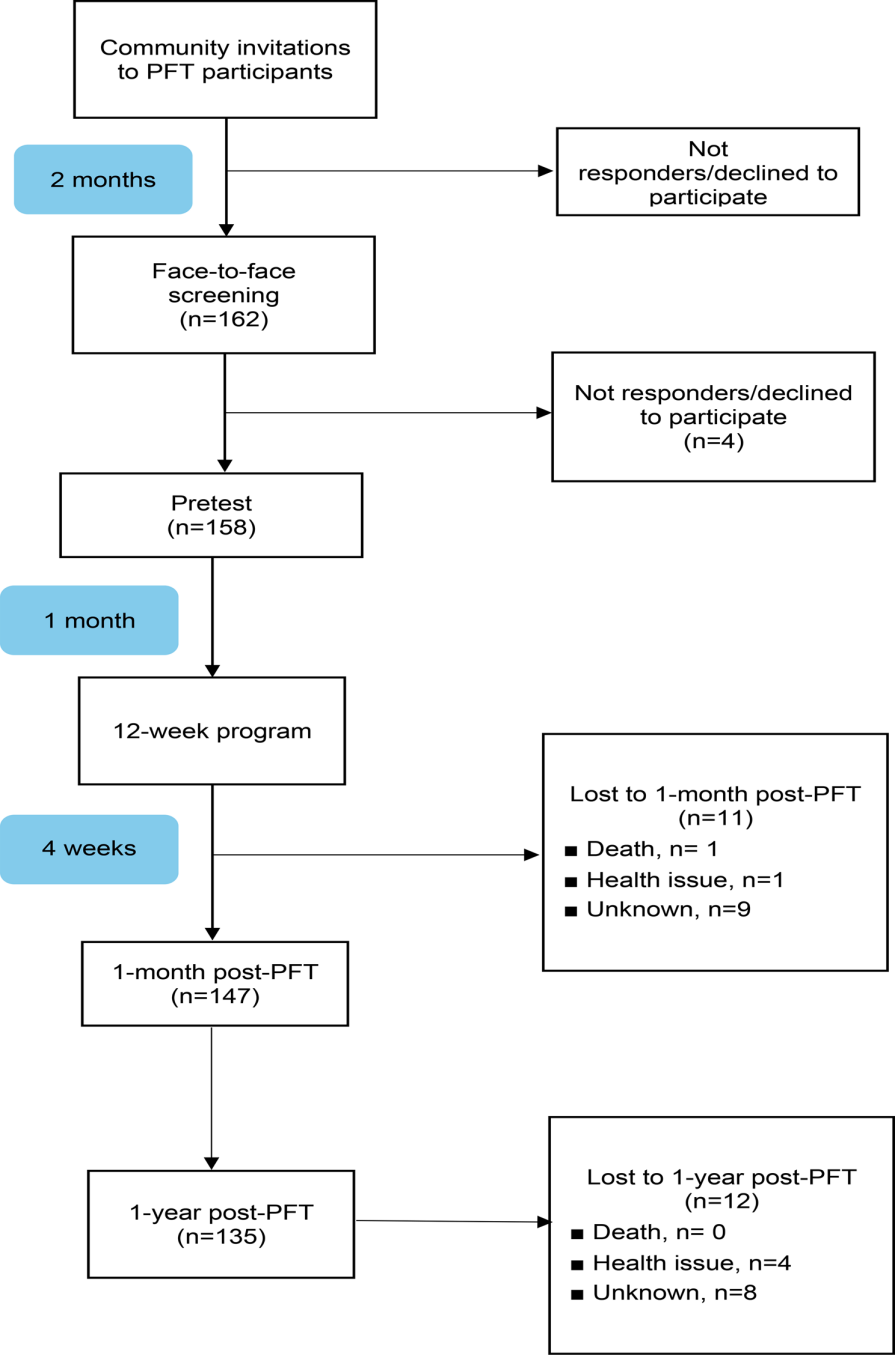
CONSORT flow diagram of participants

Table 1 displays the baseline participant characteristics. We enrolled 158 participants, of whom 74.1% (*n* = 117) were female. The average age was 78.3 years (standard deviation [SD] = 7.2). On average, participants attended 83% of the total classes. Figure 2 presents the CONSORT flow diagram of PFT study participants. Regarding living and walking status, 18.4% reported using a walking device, and 15.2% had experienced falls within the past year. The mean gait speed was 0.95 m/s (SD = 0.35), slightly below the norm of 1.0 m/s, indicating some degree of mobility impairment. Additionally, the average time for the TUG test was 14.1 s (SD = 9.0).

Age, sex, health-promoting lifestyle, community walking, and comorbidity served as control variables in statistical regression models. Approximately 46% of the participants lived on the first floor (mostly single-story buildings without stairs), and 27.4% lived alone. The average frequency of community walking was 3.84 times per week, with health-promoting lifestyle scores ranging from 24 to 69, averaging 46.89.

**Table 1:** Demographic and health characteristics at baseline (*n* = 158)

Variable	Timepoint 0 (baseline)
	Mean ± SD; *n* (%)
Female	117 (74.05%)
Age	78.34 ± 7.20
# of cohabitant members	1.98 ± 2.17
Attendance rate	0.83 ± 0.31
Cognitive impairment	54 (34.18%)
Living alone	43 (27.39%)
Housing (1st floor)	72 (45.86%)
Indoor use of walking assistive device	29 (18.35%)
Number of comorbid illnesses (0–15)	2.30 ± 1.66
Health-promoting lifestyle (24–96)	46.89 ± 10.01
ADL (0–100)	97.03 ± 6.26
IADL (0–8)	7.23 ± 1.51
Pain level within 1 month (0–10)	1.25 ± 2.54
Mini-Cog (0–5)	3.01 ± 1.56
Frequency of weekly community walking	3.84 ± 3.09
Fall occurrence within 1 year	24 (15.19%)
Fall occurrence within 1 month	8 (5.19%)
Gait speed (meter/sec)	0.95 ± 0.35
Cadence (steps/min)	113.21 ± 18.81
Gait cycle time (sec)	1.03 ± 1.19
Step length (meter)	0.54 ± 0.21
Push ratio (%)	28.59 ± 8.60
Double support (%)	25.49 ± 8.93
TUG (second)	14.07 ± 9.02
Knee extension strength (kg-force)	8.01 ± 4.76

*Abbreviations* SD, standard deviation; ADL, activities of daily living; IADL, instrumental activities of daily living; TUG, timed up and go test

### Potential impacts of PFT program

Individuals were followed up for 1 year. The community care station PFT program demonstrated significant gains in mobility among the older participants when comparing pretest and posttest at 1 month. Tables 2, 3 and 4 summarize the parameters derived from linear mixed-effects regression modeling of cognitive status and walking performance, and logit models of fall occurrence. After controlling for the factors found to be significant in the literature (i.e., age, sex, and health conditions), several variables showed significant overall positive pre/post changes.

### Cognitive function

The analysis revealed that, overall, higher age (Est = -0.06, *p* < 0.001) and greater engagement in a health-promoting lifestyle (Est = 0.02, *p* < 0.05) were associated with a significant increase in cognitive scores. Cognitive function improved significantly at 1-month post-PFT (Est = 0.25, *p* < 0.05); however, this improvement was not maintained at 1-year post-PFT.

### TUG test

Age (Est = 0.26, *p* < 0.001), living alone (Est = 2.11, *p* < 0.05), and use of an assistive walking device (Est = 2.68, *p* < 0.01) were associated with a significant increase in TUG test time, indicating worse balance performance. Health-promoting lifestyle showed a significant reduction in TUG time (Est = 0.02, *p* < 0.05). The PFT program did not have any significant short- and long-term impact on TUG performance.

### Knee extension strength

Knee extension strength did not significantly improve following the PFT program. In addition, variables such as living status (including living alone and residing in first-story housing), cognitive function, and health-promoting lifestyle were not associated with knee extension strength. However, younger individuals were more likely to have higher knee extension strength (Est = -0.17, *p* < 0.05). The knee extension strength relative to body weight (kg/kg) remained relatively low, with values ranging from 0.13 to 0.15.

### ADL

Overall, individuals with more engagement in health-promoting lifestyles (Est = 0.08, *p* < 0.01) tended to have higher ADL scores, while being female (Est = -1.53, *p* < 0.05) and using an assistive walking device (Est = -5.7, *p* < 0.001) were associated with significantly lower ADL scores. As the PFT program incorporates activities related to functional ability in daily living, we further investigated how the program improved ADL for those using walking devices with an interaction term between time and use of walking devices (walking device*time). The results showed that individuals using a walking device experienced an increase in ADL at 1-month post PFT compared with baseline (β = 2.96, *p* < 0.05). However, the ADL at 1-year post PFT did not show any differences.

**Table 2:** Unstandardized parameters resulting from linear mixed-effects regression models for cognitive function, TUG, knee extension strength, and ADL

	Cognitive function (Mini-Cog score)		TUG (seconds)		Knee extension strength (kg)		ADL	
	Est. (SE)	CL (lower, upper)	Est. (SE)	CL (lower, upper)	Est (SE)	CL (lower, upper)	Est (SE)	CL (lower, upper)
Intercept	2.71 (2.12)	-1.48, 6.90	-3.25 (7.55)	-18.17, 11.67	19.55 (11.53)	-3.24, 42.34	82.05 (5.78)***	70.64, 93.47
Age	-0.06 (0.02)***	-0.09, -0.03	0.26 (0.07)***	0.12, 0.41	-0.17 (0.08)*	-0.33, -0.02	0.08 (0.05)	-0.02, 0.18
Sex (female)	-0.43 (0.23)	-0.88, 0.02	0.15 (1.08)	-1.99, 2.28	-1.25 (1.17)	-3.57, 1.06	-1.53 (0.72)*	-2.95, -0.11
Living alone	0.17 (0.20)	-0.23, 0.57	2.11 (0.93)*	0.27, 3.95	-0.80 (1.05)	-2.87, 1.28	-0.80 (0.64)	-2.06, 0.46
Pain	-0.17 (0.17)	-0.50, 0.16	-0.17 (0.72)	-1.60, 1.27	-2.02 (1.09)	-4.18, 0.14	-0.66 (0.63)	-1.90, 0.57
Health-promoting lifestyle	0.02 (0.01)*	0.00, 0.04	-0.11 (0.04)*	-0.19, -0.02	-0.00 (0.05)	-0.10, 0.10	0.08 (0.03)**	0.02, 0.14
Mini-Cog < 3			1.32 (0.76)	-0.18, 2.82	-0.41 (1.07)	-2.53, 1.71	-0.24 (0.61)	-1.45, 0.96
Use of walking device	0.32 (0.24)	-0.15, 0.80	2.68 (1.02)**	0.67, 4.69	-0.65 (1.42)	-3.46, 2.15	-5.70 (1.14)***	-7.94, -3.45
1-month post PFT	0.25 (0.12)*	0.01, 0.50	-0.08 (0.52)	-1.10, 0.95	1.19 (0.88)	-0.55, 2.92	-0.92 (0.59)	-2.08, 0.24
1-year post PFT	0.28 (0.18)	-0.08, 0.64	-1.20 (0.77)	-2.72, 0.32	0.07 (1.19)	-2.27, 2.41	0.14 (0.65)	-1.15, 1.42
Use of walking device × T1							2.96 (1.40)*	0.21, 5.71
Use of walking device × T2							2.43 (1.37)	-0.26, 5.12

The models were adjusted for self-perceived health, comorbidity, and community walking

*Abbreviations* Est, estimate; SE, standard error; CL, confidence limit; TUG, timed up and go test; ADL, activities of daily living

* p < 0.05 ** p < 0.01 *** p < 0.001

### Gait performance

Six temporal and spatial gait parameters were analyzed to assess changes in gait performance (Table 3). The temporal parameters included gait speed, gait cycle time, and cadence, while the spatial parameters included step length, double support, and push ratio. The following sections detail the comparisons with baseline and the associations of variables with each parameter.

### Post-PFT assessments

At 1-month post-PFT, significant improvements were observed across temporal gait parameters. Gait speed increased (Est = 0.06, *p* < 0.01), gait cycle time improved (Est = 0.04, *p* < 0.01), and cadence increased (Est = 0.01, *p* < 0.05). Push ratio showed a marginally significant increase at 1-month post-PFT (Est = 1.61, *p* = 0.05). Although step length and double-support ratio showed slight improvements, they were not statistically significant. These results suggest enhanced walking ability and stability in participants shortly after completing the training program.

However, at 1-year post-PFT, these improvements were not sustained, with no significant differences observed in gait speed, gait cycle time, cadence, or step length compared with the baseline. This indicates that the positive effects of the PFT program on gait performance may diminish without continued intervention.

### Associations with gait parameters

Associations with gait parameters were analyzed while adjusting for self-perceived health, comorbidity, and frequency of community walking. All gait parameters were highly associated with the TUG test. In gait speed, higher age (Est = -0.01, *p* < 0.05) and the use of a walking device (Est = -0.08, *p* < 0.05) were associated with slower speeds. Higher knee extension strength (Est = 0.00, *p* < 0.01) and shorter TUG times (Est = -0.02, *p* < 0.001) were linked to faster gait speeds. In gait cycle time, female sex was associated with a shorter duration (Est = -0.07, *p* < 0.05), while longer TUG times were linked to longer durations (Est = 0.01, *p* < 0.001). In cadence, female participants (Est = 5.39, *p* < 0.05) and those with higher knee extension strength (Est = 0.29, *p* < 0.05) tended have higher scores. TUG times were associated with lower cadence (Est = -1.06, *p* < 0.001).

In spatial parameters, female participants exhibited an increased push ratio (Est = 3.39, *p* < 0.01), indicating greater force generation during the push-off phase of gait. The double-support ratio, expressed as a percentage of cycle duration, was significantly decreased in female participants (Est = -2.53, *p* < 0.05) and those with lower knee extension strength (Est = -0.12, *p* < 0.05), suggesting reduced balance and stability during walking.

**Fig. 3:**
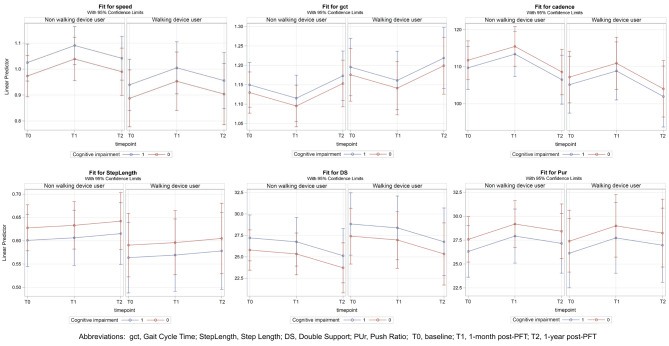
Prediction of gait parameters using walking device and cognitive status between pretest and posttest

Figure 3 illustrates the predicted changes in seven gait parameters across three time points before and after the PFT, based on walking device use and cognitive status. The gait patterns show improvements at 1-month post-PFT, with gait speed, gait cycle time, and cadence reaching statistical significance. However, these improvements returned to baseline levels by 1-year post-PFT.

**Table 3:** Unstandardized parameters resulting from linear mixed-effects regression models for gait parameter

	Speed (meter/second)	Gait cycle time (second)	Cadence (steps/minutes)	Step length (meter)	Double support (%)	Push ratio (%)
	Est (SE) CL (lower, upper)					
Intercept	1.71 (0.34)*** 1.04, 2.39	1.40 (0.24)*** 0.93, 1.88	99.19 (24.67)*** 50.45, 147.94	1.12 (0.24)*** 0.64, 1.60	26.78 (11.67)* 3.72, 49.85	33.92 (11.67)** 10.85, 56.99
Age	-0.01 (0.00)** -0.01, -0.00	-0.00 (0.00) -0.01, 0.00	-0.03 (0.18) -0.40, 0.33	-0.003 (0.00)* -0.01, -0.00	0.03 (0.08) -0.14, 0.19	-0.13 (0.08) -0.29, 0.04
Sex (female)	0.01 (0.04) -0.07, 0.08	-0.07 (0.03)* -0.12, -0.01	5.39 (2.70)* 0.05, 10.74	-0.08 (0.02)** -0.13, -0.03	-2.53 (1.18)* -4.86, -0.19	3.39 (1.2)** 1.01, 5.77
Living alone	-0.07 (0.03)* -0.13, -0.00	0.01 (0.02) -0.04, 0.06	-0.65 (2.39) -5.38, 4.07	-0.002 (0.02) -0.05, 0.04	2.00 (1.07) -0.12, 4.12	-2.77 (1.08)* -4.91, -0.63
Knee extension strength (kg)	0.00 (0.00)** 0.00, 0.01	-0.00 (0.00) -0.00, 0.00	0.29 (0.11)* 0.06, 0.51	0.002 (0.00)* 0.00, 0.00	-0.12 (0.06)* -0.24, -0.01	-0.03 (0.06) -0.14, 0.08
TUG (seconds)	-0.02 (0.00)*** -0.03, -0.02	0.01 (0.00)*** 0.01, 0.02	-1.06 (0.16)*** -1.37, -0.75	-0.01 (0.00)*** -0.01, -0.01	0.46 (0.08)*** 0.31, 0.61	-0.44 (0.08)*** -0.59, -0.29
Use of walking device	-0.08 (0.04)* -0.16, -0.00	0.05 (0.03) -0.00, 0.11	-4.87 (2.94) -10.68, 0.94	-0.04 (0.03) -0.1, 0.02	1.62 (1.43) -1.2, 4.44	-0.19 (1.41) -2.98, 2.6
Mini-Cog < 3	-0.05 (0.03) -0.11, 0.01	0.02 (0.02) -0.02, 0.06	-1.83 (2.16) -6.10, 2.44	-0.03 (0.02) -0.07, 0.02	1.42 (1.07) -0.7, 3.54	-1.26 (1.05) -3.34, 0.81
Health promotion lifestyle	0.00 (0.00) -0.00, 0.00	0.00 (0.00) -0.00, 0.00	-0.13 (0.11) -0.35, 0.09	-0.00 (0.00) -0.00, 0.00	0.05 (0.05) -0.05, 0.16	0.01 (0.05) -0.09, 0.12
1-month post PFT	0.06 (0.02)** 0.02, 0.10	-0.04 (0.01)** -0.06, -0.01	3.88 (1.55)* 0.81, 6.95	0.01 (0.02) -0.03, 0.04	-0.45 (0.87) -2.18, 1.28	1.61 (0.83) -0.03, 3.24
1-year post PFT	0.01 (0.03) -0.05, 0.07	0.02 (0.02) -0.02, 0.06	-2.82 (2.24) -7.25, 1.61	0.01 (0.03) -0.04, 0.06	-2.08 (1.23) -4.51, 0.35	0.84 (1.15) -1.43, 3.12

The models were adjusted for self-perceived health, comorbidity and community walking

*Abbreviations* Est, estimate; SE, standard error; CL, confidence limit, TUG, timed up and go test

* *p* < 0.05 ** *p* < 0.01 *** *p* < 0.001

### Falls within 1 month

Overall, living alone (Est = 1.69, *p* < 0.01) and pain (Est = 1.65, *p* < 0.05) were significantly associated with a higher probability of falls. Individuals with higher gait speed (Est = -14.47, *p* < 0.05) and ADL (Est = -0.10, *p* < 0.01) were less likely to experience falls, suggesting that better physical performance is protective. Additionally, the probabilities of falling did not significantly change 1 month or 1 year after the PFT program, indicating that the immediate and long-term post-PFT periods were not associated with an altered risk for falls (Table 4).

**Table 4:** Unstandardized parameters resulting from logit models for fall occurrence

	Fall in 1 month	
	Est (SE)	CL (lower, upper)
Intercept	34.53 (33.30)	-30.74, 99.80
Age	-0.05 (0.06)	-0.17, 0.07
Sex (female)	0.36 (1.14)	-1.88, 2.60
Comorbidity	0.21 (0.25)	-0.28, 0.70
Living alone	1.69 (0.60)**	0.51, 2.87
Perceived health status	0.07 (0.43)	-0.77, 0.90
Health promotion lifestyle	0.03 (0.05)	-0.07, 0.14
Mini-Cog < 3	-0.42 (0.79)	-1.96, 1.12
Pain	1.65 (0.79)*	0.11, 3.19
Speed	-14.47 (7.10)*	-28.38, -0.55
ADL	-0.10 (0.04)**	-0.17, -0.03
Knee extension strength	0.02 (0.03)	-0.04, 0.08
Balance	-0.01 (1.21)	-2.38, 2.37
Use of walking device	0.29 (0.79)	-1.26, 1.85
1-month post PFT	0.79 (0.76)	-0.70, 2.29
1-year post PFT	0.90 (1.47)	-1.98, 3.79

*Abbreviations* Est, estimate; CL, confidence limit; ADL, activities of daily living

* *p* < 0.05 ** *p* < 0.01 ***

## Discussion

We observed the progress of improvements in ADL, gait performance, and cognitive functions at short-term and long-term time points. This is one of the few studies to examine longitudinal mobility by walking device use in a community-based sample using multiple gait parameters. The program boosted gait performance in terms of temporal gait parameters such as gait speed, gait cycle time, and cadence. Knee extension strength and TUG test performance did not present significant improvements in the program, and the results showed the risk factors for walking ability decline and fall occurrence. Implementation of existing and developing community-based group-tailored programs for older adults with a variety of mobility difficulties may offer an innovative way to overcome these issues.

The baseline ADL and IADL scores were generally high, with an average ADL score of 97. Only one participant (0.63%) exhibited a moderate level of dependence (score < 65). The mean IADL score at baseline was 7 out of 8, with 12 participants (7.59%) requiring assistance with at least three items. ADL changes by the use of walking devices at 1-month post-PFT significantly improved. Significant improvements in cognitive function and gait parameters (speed, cadence, and gait cycle time) were observed at 1-month post-PFT, indicating the immediate positive impact of the program. However, the absence of significant changes in knee extension strength, balance, and fall occurrences suggests that while cognitive and motor skills can improve in the short term, more targeted interventions may be needed to enhance physical strength and balance. The disappearance of these improvements at the 1-year posttest raises questions about the long-term effectiveness of the training, suggesting that ongoing or repeated training may be necessary to maintain the short-term benefits.

In terms of muscle strength, the findings related to knee extension strength were not significantly improved following the PFT program, suggesting that knee extension strength may not fully capture the impact of physical training within a 1-month period and may not sustain improvements over a year without continued training during that time. Further analysis revealed that cognitive function and health lifestyle factors were not significantly associated with knee extension strength. However, we did find that age was significantly associated with knee extension strength (Est = -0.17, *p* < 0.05), highlighting the natural decline in knee extension strength with advancing age. The low knee extension strength values relative to body weight (0.13 to 0.15 kg/kg) observed in this study are concerning when compared with the range of 0.35 to 0.45 kg/kg reported in existing studies (19, 21), as they suggest a greater risk for functional limitations and mobility impairments in this population. This further reveals the need for targeted interventions that can effectively address and mitigate age-related muscle decline.

In this study, the observed cadence of 113.21 steps/min aligns closely with the literature, which reports 114.74 steps/min [57]. The gait speed measured at 0.95 m/second falls within the broader range of 0.8–1.46 m/second documented in prior research [57–61]. The incidence of falls, at 15.19%, is marginally lower than the 19.1–21% 1-year prevalence reported in Taiwanese studies [7, 15]. Identified risk factors for falls include living alone, experiencing pain, and scoring low on mobility function assessments. Gait speed, serving as a clinical indicator of mobility, exhibited a short-term improvement in response to the PFT program.

Two types of gait phases were obtained in this study (push ratio and double support). The double-support phase is considered the most stable period, as one limb prepares to push off the ground while the other bears the full weight of the body [62, 63]. As double-support percentage refers to the phase of the gait cycle when both feet are in contact with the ground, an increase in double support may reflect the greater effort needed to maintain stability while walking [63]. Although the quality and standards of the gait phase ratios are not yet comprehensively understood, the observed gait patterns could still provide some insights. Double-support percentages typically range from 24.5 to 26.6% [63]. The double-support percentage in this study (25.49%) falls within that range. Though the post-PFT assessments showed a decrease in double support, the change was not statistically significant. Although not all gait parameters showed statistically significant improvements, the overall trends suggest that the PFT program had a positive impact on gait dynamics. These changes suggest that the program influenced the way participants walk, potentially improving mobility. Even though some parameters did not reach statistical significance, the alterations in gait patterns provide valuable insights into how functional training can modify walking mechanics over time.

In this study, older adults with more engagement in health-promoting lifestyles were more likely to have better cognitive function and balance performance. Additionally, the impacts of living alone and use of walking devices on TUG scores, knee extension strength, gait speed, and fall risk were revealed, emphasizing the importance of a holistic approach to geriatric care, which considers not just physical training but also living conditions and the appropriate use of assistive technologies. Our findings also indicated that individuals using a walking device may have particularly greater short-term improvements in ADL by the PFT. However, reduction of fall occurrence, TUG, and knee extension strength did not reveal a significant longitudinal change over time.

The PFT program was designed to enhance functional capacities in older adults, focusing on walking and cognitive function, both critical for daily activities. The data revealed a predominance of females (74.1%) among participants, reflecting the demographic trends in older age groups. In addition, 27.4% of the participants lived alone, which can lead to health challenges such as isolation, reduced social support, and a lack of emergency assistance. To sustain improvements over time, intervention programs for community-dwelling older adults may require continuous or periodic reinforcement sessions. These programs should also consider individuals’ living situation and the judicious use of walking devices to tailor interventions effectively. Healthcare providers or community coordinators should be aware of the risk factors associated with living alone and the potential for reduced mobility and increased fall risk, suggesting a need for community and social support mechanisms.

Future programs should prioritize personalized strategies, particularly for those living alone, and integrate cognitive exercises into functional training. Tailored approaches, dynamic feedback, and participant education will be essential for long-term success. By focusing on individual mobility needs, these interventions can enhance the overall well-being of older adults in community settings.

### Study limitations

This study offers key insights into the impacts of community-based training programs on walking and cognitive abilities in older adults. However, several limitations exist, including the absence of a control group, a small sample size potentially reducing generalizability, potential recall bias from self-reported fall histories, and health characteristics affecting broader applicability. In the future, these limitations should be addressed by conducting large-scale controlled studies with extended follow-up periods. Diverse mobility measures, ranging from balance to dual-task performance, would provide a holistic evaluation. It is also important to consider psychological factors, such as fear of falling and quality of life, both of which are crucial for older adults’ mobility. Refining these areas will shape the design and application of programs that optimize the mobility and health of older populations.

It is important to recognize that the impact of stairs on mobility can vary widely depending on individual health, physical abilities, and the environment. When assessing mobility in a research or healthcare context, it is crucial to consider the specific challenges posed by stairs and their potential influence on an individual’s ability to move independently and safely.

## Conclusion

This study demonstrates that a 12-week community-based PFT program may lead to short-term improvements in cognitive function, ADL, and gait performance in older adults. The findings support broader implementation through regional and national agencies, enhancing the impact of functional training in communities. The 1-year follow-up data offer valuable insights into ADL tasks, gait performance, and fall risk, allowing for more accurate predictions of functional training’s long-term effects. Furthermore, the gait analysis can provide a better understanding of the measurement perspective when evaluating the effectiveness of training programs aimed at improving walking.

## Data Availability

The data that support the findings of this study were collected by the research team. But restrictions apply to the availability of these data, which were used under license for the current study, and so are not publicly available.

## Abbreviations

PFT: Precision Functional Training
TUG: Timed Up-and-Go
ADL: Activities of Daily Living
RPE: Rated Perceived Exertion
SD: Standard Deviation
CONSORT: Consolidated Standards of Reporting Trials

## References

[1] National Development Council. Population projections for the R.O.C. Taiwan 2024. https://www.ndc.gov.tw/Content_List.aspx?n=2688C8F5935982DC. Accessed 25 September, 2024.

[2] Lin PS, Hsieh CC, Cheng HS, Tseng TJ, Su SC. Association between physical fitness and successful aging in Taiwanese older adults. PLoS ONE. 2016;11(3):e0150389. 26963614 10.1371/journal.pone.0150389 PMC4786127

[3] Tinetti ME, Kumar C. The patient who falls: it’s always a trade-off. JAMA. 2010;303(3):258–66. 20085954 10.1001/jama.2009.2024 PMC3740370

[4] Cruz-Jimenez M. Normal changes in gait and mobility problems in the elderly. Phys Med Rehabil Clin N Am. 2017;28(4):713–25. 29031338 10.1016/j.pmr.2017.06.005

[5] Alexander BH, Rivara FP, Wolf ME. The cost and frequency of hospitalization for fall-related injuries in older adults. Am J Public Health. 1992;82(7):1020–3. 1609903 10.2105/ajph.82.7.1020 PMC1694056

[6] Studenski S, Perera S, Patel K, Rosano C, Faulkner K, Inzitari M, et al. Gait speed and survival in older adults. JAMA. 2011;305(1):50–8. 21205966 10.1001/jama.2010.1923 PMC3080184

[7] Kuo FL, Yen CM, Chen HJ, Liao ZY, Lee Y. Trajectories of mobility difficulty and falls in community-dwelling adults aged 50 + in Taiwan from 2003 to 2015. BMC Geriatr. 2022;22(1):902. 36434511 10.1186/s12877-022-03613-3 PMC9700940

[8] Tsai LT, Rantakokko M, Portegijs E, Viljanen A, Saajanaho M, Eronen J, et al. Environmental mobility barriers and walking for errands among older people who live alone vs. with others. BMC Public Health. 2013;13(1):1–8. 24207063 10.1186/1471-2458-13-1054 PMC4226209

[9] Chang SH, Hsueh MC, Liao Y. Personal and behavioral correlates of total and domain-specific sedentary behaviors in older Taiwanese adults. BMC Geriatr. 2018;18(294):1–7. 30497416 10.1186/s12877-018-0987-9 PMC6267834

[10] King DB, Yoon JY, Pecanac K, Brown R, Mahoney J. Frequency and duration of nursing care related to older patient mobility. J Nurs Scholarsh. 2014;46(1):20–7. 24112775 10.1111/jnu.12047

[11] Craig J, Murray A, Mitchell S, Clark S, Saunders L, Burleigh L. The high cost to health and social care of managing falls in older adults living in the community in Scotland. Scott Med J. 2013;58(4):198–203. 24215036 10.1177/0036933013507848

[12] Close JC, Lord SR, Antonova EJ, Martin M, Lensberg B, Taylor M, et al. Older people presenting to the emergency department after a fall: a population with substantial recurrent healthcare use. Emerg Med J. 2012;29(9):742–7. 21965178 10.1136/emermed-2011-200380

[13] Stevens JA, Thomas K, Teh L, Greenspan AI. Unintentional fall injuries associated with walkers and canes in older adults treated in U.S. emergency departments. J Am Geriatr Soc. 2009;57(8):1464–9. 19555423 10.1111/j.1532-5415.2009.02365.x

[14] Scheffer AC, Schuurmans MJ, Van Dijk N, Van Der Hooft T, De Rooij SE. Fear of falling: measurement strategy, prevalence, risk factors and consequences among older persons. Age Ageing. 2008;37(1):19–24. 18194967 10.1093/ageing/afm169

[15] Chen PL, Lin HY, Ong JR, Ma HP. Development of a fall-risk assessment profile for community-dwelling older adults by using the National Health Interview Survey in Taiwan. BMC Public Health. 2020;20(1):234. 32059657 10.1186/s12889-020-8286-8 PMC7023681

[16] Liao Y, Hsu HH, Shibata A, Ishii K, Koohsari MJ, Oka K. Associations of total amount and patterns of objectively measured sedentary behavior with performance-based physical function. Prev Med Rep. 2018;12:128–34. 30234001 10.1016/j.pmedr.2018.09.007 PMC6139483

[17] Studenski S, Perera S, Wallace D, Chandler JM, Duncan PW, Rooney E, et al. Physical performance measures in the clinical setting. J Am Geriatr Soc. 2003;51(3):314–22. 12588574 10.1046/j.1532-5415.2003.51104.x

[18] Shumway Cook A, Brauer S, Woollacott M. Predicting the probability for falls in community-dwelling older adults using the timed up & go test. Phys Ther. 2000;80(9):896–903. 10960937

[19] Martien S, Delecluse C, Boen F, Seghers J, Pelssers J, Van Hoecke A-S, et al. Is knee extension strength a better predictor of functional performance than handgrip strength among older adults in three different settings? Arch Gerontol Geriatr. 2015;60(2):252–8. 25496605 10.1016/j.archger.2014.11.010

[20] Yeung SS, Reijnierse EM, Trappenburg MC, Blauw GJ, Meskers CG, Maier AB. Knee extension strength measurements should be considered as part of the comprehensive geriatric assessment. BMC Geriatr. 2018;18:1–8. 29859054 10.1186/s12877-018-0815-2 PMC5984755

[21] Kojima N, Kim H, Saito K, Yoshida H, Yoshida Y, Hirano H, et al. Association of knee-extension strength with instrumental activities of daily living in community‐dwelling older adults. Geriatr Gerontol Int. 2014;14(3):674–80. 24215603 10.1111/ggi.12158

[22] Simonsick EM, Guralnik JM, Fried LP. Who walks? Factors associated with walking behavior in disabled older women with and without self-reported walking difficulty. J Am Geriatr Soc. 1999;47(6):672–80. 10366165 10.1111/j.1532-5415.1999.tb01588.x

[23] Thomas VS, Vandenberg EV, Potter JF. Non-neurological factors are implicated in impairments in gait and mobility among patients in a clinical dementia referral population. Int J Geriatr Psychiatry. 2002;17(2):128–33. 11813274 10.1002/gps.547

[24] Chen ST, Stevinson C, Tian T, Chen LJ, Ku PW. Accelerometer-measured daily steps and subjective cognitive ability in older adults: a two-year follow-up study. Exp Gerontol. 2020;133:110874. 32057824 10.1016/j.exger.2020.110874

[25] Yang Y, Wang K, Liu H, Qu J, Wang Y, Chen P, et al. The impact of Otago exercise programme on the prevention of falls in older adult: a systematic review. Front Public Health. 2022;10:953593. 36339194 10.3389/fpubh.2022.953593 PMC9631473

[26] Yogev-Seligmann G, Hausdorff JM, Giladi N. The role of executive function and attention in gait. Mov Disord. 2008;23(3):329–42. 18058946 10.1002/mds.21720 PMC2535903

[27] Gallou Guyot M, Mandigout S, Combourieu Donnezan L, Bherer L, Perrochon A. Cognitive and physical impact of cognitive-motor dual-task training in cognitively impaired older adults: an overview. Neurophysiol Clin. 2020;50(6):441–53. 33121880 10.1016/j.neucli.2020.10.010

[28] Aoyama M, Suzuki Y, Onishi J, Kuzuya M. Physical and functional factors in activities of daily living that predict falls in community-dwelling older women. Geriatr Gerontol Int. 2011;11(3):348–57. 21265970 10.1111/j.1447-0594.2010.00685.x

[29] Sherrington C, Fairhall N, Kwok W, Wallbank G, Tiedemann A, Michaleff ZA, et al. Evidence on physical activity and falls prevention for people aged 65 + years: systematic review to inform the WHO guidelines on physical activity and sedentary behaviour. Int J Behav Nutr Phys Act. 2020;17(1):144. 33239019 10.1186/s12966-020-01041-3 PMC7689963

[30] Lee SH, Yu S. Effectiveness of multifactorial interventions in preventing falls among older adults in the community: a systematic review and meta-analysis. Int J Nurs Stud. 2020;106:103564. 32272282 10.1016/j.ijnurstu.2020.103564

[31] Yu HW, Tu YK, Chen YM. Sociodemographic characteristics, disability trajectory, and health care and long-term care utilization among middle-old and older adults in Taiwan. Arch Gerontol Geriatr. 2019;82:161–6. 30802840 10.1016/j.archger.2019.01.019

[32] Fien S, Linton C, Mitchell JS, Wadsworth DP, Szabo H, Askew CD, et al. Characteristics of community-based exercise programs for community-dwelling older adults in rural/regional areas: a scoping review. Aging Clin Exp Res. 2022;34(7):1511–28. 35152393 10.1007/s40520-022-02079-y PMC8852913

[33] Chan SY, Kuo CC, Chen KM, Tseng WS, Huang HT, Li CH. Health promotion outcomes of a newly developed elastic band exercise program for older adults in the community: a pilot test. J Nurs Res. 2016;24(2):137–44. 26258390 10.1097/jnr.0000000000000099

[34] Ho HC, Lin HY, Tai PF, Ho HL. Effects of community participation on the psychological well-being of the elderly: a case study of the community caring concern centers in Pingtung County. Taiwan Association Community Work Study. 2016;6(3):45–80.

[35] Chuang YF, Liu YC, Tseng HY, Lin PX, Li CY, Shih MH, et al. Urban-rural differences in the prevalence and correlates of mild cognitive impairment in community-dwelling older adults in Taiwan: the EMCIT study. J Formos Med Assoc. 2021;120(9):1749–57. 33810927 10.1016/j.jfma.2021.03.005

[36] Huang CY, Lee WJ, Lin HP, Chen RC, Lin CH, Peng LN, et al. Epidemiology of frailty and associated factors among older adults living in rural communities in Taiwan. Arch Gerontol Geriatr. 2020;87:103986. 31901458 10.1016/j.archger.2019.103986

[37] Department of Household Registration Taiwan. Demographics 2023. https://www.ris.gov.tw/app/portal/346. Accessed 25 September, 2024.

[38] Borg GA. Psychophysical bases of perceived exertion. Med Sci Sports Exerc. 1982;14(5):377–81. 7154893

[39] Paraschiv Ionescu A, Büla CJ, Major K, Lenoble Hoskovec C, Krief H, El Moufawad C, et al. Concern about falling and complexity of free-living physical activity patterns in well-functioning older adults. Gerontology. 2018;64(6):603–11. 29972821 10.1159/000490310 PMC6262680

[40] Trombini Souza F, de Maio Nascimento M, da Silva TFA, de Araújo RC, Perracini MR, Sacco Isabel CN. Dual-task training with progression from variable-to fixed-priority instructions versus dual-task training with variable-priority on gait speed in community-dwelling older adults: a protocol for a randomized controlled trial: variable-and fixed-priority dual-task for older adults. BMC Geriatr. 2020;20:1–12. 10.1186/s12877-020-1479-2 PMC7036177 32087694

[41] Allet L, Armand S, de Bie RA, Golay A, Monnin D, Aminian K, et al. Reliability of diabetic patients’ gait parameters in a challenging environment. Gait Posture. 2008;28(4):680–6. 18579384 10.1016/j.gaitpost.2008.05.006

[42] de Bruin E, Najafi B, Murer K, Uebelhart D, Aminian K. Quantification of everyday motor function in a geriatric population. J Rehabil Res Dev. 2007;44(3):417–28. 18247238 10.1682/jrrd.2006.01.0003

[43] Hsu CL, Liang CK, Liao MC, Chou MY, Lin YT. Slow gait speed as a predictor of 1-year cognitive decline in a veterans’ retirement community in southern Taiwan. Geriatr Gerontol Int. 2017;17:14–9. 28436187 10.1111/ggi.13034

[44] Jerome GJ, Ko SU, Kauffman D, Studenski SA, Ferrucci L, Simonsick EM. Gait characteristics associated with walking speed decline in older adults: results from the Baltimore Longitudinal Study of Aging. Arch Gerontol Geriatr. 2015;60(2):239–43. 25614178 10.1016/j.archger.2015.01.007 PMC4330111

[45] Podsiadlo D, Richardson S. The timed up & go: a test of basic functional mobility for frail elderly persons. J Am Geriatr Soc. 1991;39(2):142–8. 1991946 10.1111/j.1532-5415.1991.tb01616.x

[46] Barry E, Galvin R, Keogh C, Horgan F, Fahey T. Is the timed up and go test a useful predictor of risk of falls in community dwelling older adults: a systematic review and meta-analysis. BMC Geriatr. 2014;14:14. 24484314 10.1186/1471-2318-14-14 PMC3924230

[47] Savva GM, Donoghue OA, Horgan F, O’Regan C, Cronin H, Kenny RA. Using timed up-and-go to identify frail members of the older population. J Gerontol Biol Sci Med Sci. 2012;68(4):441–6. 10.1093/gerona/gls190 22987796

[48] Liao CD, Chen HC, Huang SW, Liou TH. The role of muscle mass gain following protein supplementation plus exercise therapy in older adults with Sarcopenia and frailty risks: a systematic review and meta-regression analysis of randomized trials. Nutrients. 2019;11(8):1713. 31349606 10.3390/nu11081713 PMC6723070

[49] Salem GJ, Wang MY, Young JT, Marion M, Greendale GA. Knee strength and lower- and higher-intensity functional performance in older adults. Med Sci Sports Exerc. 2000;32(10):1679–84. 11039637 10.1097/00005768-200010000-00003

[50] Mentiplay BF, Perraton LG, Bower KJ, Adair B, Pua YH, Williams GP, et al. Assessment of lower limb muscle strength and power using hand-held and fixed dynamometry: a reliability and validity study. PLoS ONE. 2015;10(10):e0140822. 26509265 10.1371/journal.pone.0140822 PMC4624940

[51] Rubenstein LZ. Falls in older people: epidemiology, risk factors and strategies for prevention. Age Ageing. 2006;35(Suppl 2):ii37–41. 16926202 10.1093/ageing/afl084

[52] Wade DT, Collin C. The Barthel ADL index: a standard measure of physical disability? Int Disabil Stud. 1988;10(2):64–7. 3042746 10.3109/09638288809164105

[53] Graf C. Functional decline in hospitalized older adults: it’s often a consequence of hospitalization, but it doesn’t have to be. Am J Nurs. 2006;106(1):58–67. 16481783 10.1097/00000446-200601000-00032

[54] Lawton MP, Brody EM. Assessment of older people: self-maintaining and instrumental activities of daily living. Gerontologist. 1969;9(3):179–86. 5349366

[55] Borson S, Scanlan JM, Chen P, Ganguli M. The Mini-cog as a screen for dementia: validation in a population-based sample. J Am Geriatr Soc. 2003;51(10):1451–4. 14511167 10.1046/j.1532-5415.2003.51465.x

[56] Wei M, Lu C. Development of the short-form Chinese Health-promoting Lifestyle Profile. J Health Edu. 2005;24:25–46.

[57] Bilney B, Morris M, Webster K. Concurrent related validity of the GAITRite^®^ walkway system for quantification of the spatial and temporal parameters of gait. Gait Posture. 2003;17(1):68–74. 12535728 10.1016/s0966-6362(02)00053-x

[58] Kawai H, Taniguchi Y, Seino S, Sakurai R, Osuka Y, Obuchi S, et al. Reference values of gait parameters measured with a plantar pressure platform in community-dwelling older Japanese adults. Clin Interv Aging. 2019;14:1265–76. 31371932 10.2147/CIA.S213216 PMC6636431

[59] Chen LK, Woo J, Assantachai P, Auyeung TW, Chou MY, Iijima K, et al. Asian Working Group for Sarcopenia: 2019 consensus update on Sarcopenia diagnosis and treatment. J Am Med Dir Assoc. 2020;21(3):300–e72. 32033882 10.1016/j.jamda.2019.12.012

[60] Lin YH, Chen HC, Hsu NW, Chou P. Using hand grip strength to detect slow walking speed in older adults: the Yilan study. BMC Geriatr. 2021;21(1):428. 34271880 10.1186/s12877-021-02361-0 PMC8285830

[61] Wu IC, Lin CC, Hsiung CA, Wang CY, Wu CH, Chan DC, et al. Epidemiology of Sarcopenia among community-dwelling older adults in Taiwan: a pooled analysis for a broader adoption of sarcopenia assessments. Geriatr Gerontol Int. 2014;14(S1):52–60. 24450561 10.1111/ggi.12193

[62] Winter DA, Patla AE, Frank JS, Walt SE. Biomechanical walking pattern changes in the fit and healthy elderly. Phys Ther. 1990;70(6):340–7. 2345777 10.1093/ptj/70.6.340

[63] Kwon MS, Kwon YR, Park YS, Kim JW. Comparison of gait patterns in elderly fallers and non-fallers. Technol Health Care. 2018;26(S1):427–36. 29758966 10.3233/THC-174736 PMC6004957

